# Pseudobulbar Affect: A Network Disorder Linking Emotion, Neurobiology, and Therapeutics

**DOI:** 10.7759/cureus.96829

**Published:** 2025-11-14

**Authors:** Beatriz De Faria Sousa, Juan B Espinosa

**Affiliations:** 1 College of Medicine, Florida International University, Herbert Wertheim College of Medicine, Miami, USA; 2 Psychiatry, Memorial Regional Hospital, Hollywood, USA; 3 Psychiatry, Florida International University, Herbert Wertheim College of Medicine, Miami, USA

**Keywords:** cortico-limbic-pontocerebellar network, dextromethorphan-quinidine, emotional dysmetria, emotional incontinence, glutamatergic neurotransmission, neuropsychiatry, pathological laughing and crying, pseudobulbar affect, serotonergic dysfunction

## Abstract

Pseudobulbar affect (PBA), historically described as emotional incontinence, is a neurologic syndrome characterized by sudden, involuntary episodes of laughing or crying that are disproportionate to context and incongruent with mood. Once viewed as a minor behavioral complication of brain injury, it is now understood as a network-level disorder of emotional expression, arising from disinhibition of the brainstem faciovocal apparatus rather than from alterations in emotional experience.

This narrative review synthesizes 39 peer-reviewed studies, examining the historical origins, pathophysiology, clinical recognition, and treatment of PBA across neurologic diseases. Classical descriptions by Magnus, Bechterew, and Wilson anticipated contemporary findings, that disruption of cortico-limbic-pontocerebellar circuits, together with neurotransmitter imbalance in serotonergic and glutamatergic pathways, underlie these episodes. Modern neuroimaging and electrophysiologic evidence demonstrate that structural and functional connectivity loss within this network leads to dysregulation of affective output, or emotional dysmetria. Validated instruments, such as the Center for Neurologic Study-Lability Scale (CNS-LS), improve recognition, while pharmacologic therapies, including selective serotonin reuptake inhibitors (SSRIs) and dextromethorphan/quinidine (DM/Q), restore inhibitory balance. Conceptualizing PBA as a disorder of expressive control rather than affective tone reframes it as a treatable manifestation of network dysfunction. Future directions include biomarker development, cerebellar-targeted neuromodulation, and integration of standardized screening into neurologic care.

## Introduction and background

Pseudobulbar affect (PBA) presents a diagnostic challenge because its paroxysmal emotional outbursts resemble mood disorders, while arising from distinct neural mechanisms. These brief, uncontrollable fits of laughter or crying represent transient failures in the regulation of emotional expression, rather than sustained changes in mood. Although classically described in amyotrophic lateral sclerosis (ALS) and multiple sclerosis (MS), PBA also occurs following stroke, traumatic brain injury (TBI), and cerebellar lesions [1-3]. Beyond the neurologic context, its psychosocial repercussions - embarrassment, withdrawal, and misdiagnosis as depression - can be profound [4].

While contemporary research emphasizes network dysfunction and neurotransmitter imbalance, early neurologists such as Magnus and Bechterew conceptualized these episodes as a loss of voluntary control over the faciovocal apparatus - the muscles that express laughter and crying. This historical foundation informs modern understanding of PBA as a disorder of emotional expression rather than emotional experience, a distinction that underlies its modern neurobiological interpretation.

Historical and conceptual definition

The syndrome now widely termed PBA, also historically known as pathological laughing and crying, involuntary emotional expression disorder, or most precisely emotional incontinence, has long been recognized as a disorder of emotional expression, not of mood itself. In 1837, Adolf Magnus described a patient who lost voluntary control of crying and laughter following multiple cerebral infarcts, noting that the emotional displays were disconnected from the patient’s inner feeling [5]. Wladimir Bechterew later expanded on this idea, coining the expression “incontinence of laughter and crying in affections of the brain” and drawing a parallel to urinary incontinence to emphasize an inability to suppress automatic outflow once the neural “sphincter” of emotional control is lost [6]. He proposed that the faciovocal apparatus - the network of facial, bulbar, respiratory, and laryngeal muscles responsible for laughter, crying, and other affective expressions - is normally under inhibitory modulation from higher cortical centers [6].

Building on this framework, Kinnier Wilson (1924) proposed a supranuclear pontobulbar center for affective synergisms: a brainstem integration hub coordinating the synchronized activation of facial, respiratory, and phonatory components of emotional expression [7]. Under normal conditions, corticobulbar and limbic inputs maintain tonic inhibition over this center. When that modulation is interrupted by disease or injury, the center discharges automatically, producing involuntary laughter or crying, which may be incongruent with the patient’s subjective state [7]. This classical “release” model remains supported by contemporary lesion-mapping and neuroimaging data, demonstrating network-level disinhibition within cortico-limbic-pontocerebellar circuits [8]. Wilson’s model also distinguished true emotional incontinence, a failure of inhibitory gating, from emotional lability, in which authentic emotions are merely amplified but remain consciously experienced [7,8].

Modern authors reaffirm that the core feature of this syndrome is the release of the faciovocal mechanisms that express laughter or crying, without the concomitant subjective emotional experiences of mirth or sadness [9]. Patients remain capable of genuine emotion but lose executive control over its expression. While emotional incontinence most accurately conveys this phenomenon, PBA remains the prevailing clinical term and will be used in this review when discussing diagnostic instruments, neuroanatomy, and therapy, with the understanding that it denotes an expressive, not experiential, disorder. This definitional clarity provides the foundation for viewing PBA as a network-level disturbance of inhibitory-excitatory control across cortico-limbic-pontocerebellar circuits, uniting its historical phenomenology with contemporary neurobiological evidence [10].

## Review

Methods

A focused narrative review was conducted using PubMed and Google Scholar for English-language publications from 1985 to 2025, using the keywords PBA, pathophysiology, neuroanatomy, diagnosis, and treatment. Twenty-eight peer-reviewed primary sources and 11 supplemental historical and contemporary articles (a total of 39) were analyzed qualitatively. Studies included clinical trials, case reports, reviews, and imaging investigations. Findings were grouped into four domains: (1) neuroanatomical and neurochemical basis, (2) clinical recognition and differentiation, (3) therapeutic management, and (4) emerging directions.

Given the wide variation in study designs, diagnostic definitions, and outcome measures - including differences in how affective episodes were identified and quantified - a formal meta-analysis was neither feasible nor conceptually appropriate; thus, heterogeneous study designs precluded quantitative synthesis.

Pathophysiology of PBA: a network disorder

Early accounts attributed PBA to the “release phenomenon,” in which cortical damage disinhibits brainstem laughter- and crying centers [11]. However, subsequent clinico-anatomical reports have shown that the syndrome is not exclusively cortical. Patients have developed pathological laughing and crying from basal forebrain and pontine lesions, without other signs of pseudobulbar palsy, confirming that true emotional incontinence can arise from injury at multiple levels of the emotional-expression network [9]. These observations reinforce that PBA is best conceptualized as a network-level dysfunction spanning cortical, subcortical, and brainstem regions, rather than a purely supratentorial phenomenon.

Beyond neural disinhibition, PBA also features a distinctive pattern of vegetative co-activation - tearing, facial flushing, salivation, and autonomic arousal - that accompanies incontinent laughter and crying [12]. This visceral-somatic coupling reflects the close anatomical integration of brainstem autonomic nuclei with the motor circuits that control facial and vocal expression. Electromyographic studies demonstrate differential activation of facial and laryngeal muscles during these episodes, confirming a brainstem-driven, reflexive expressive pattern rather than a volitional display [12]. These physiological findings anticipated later network-based models of emotional regulation, illustrating that the expressive and autonomic components of emotion share a common neural substrate.

Contemporary neuroimaging and lesion studies have confirmed and expanded the classical “release” model, revealing a distributed cortico-limbic-pontocerebellar network that regulates the intensity and context of emotional expression [13,14]. Within this circuitry, the cerebellum plays a key role in affective modulation: its disruption, particularly of cerebellar or pontine pathways, produces emotional dysmetria, or poorly calibrated emotional output [7,8]. Functional MRI studies further demonstrate that loss of resting-state connectivity within these circuits underlies disinhibited affective expression in ALS [15], linking historical clinico-anatomical observations to modern network neuroscience.

Building on these observations, disruption of descending inhibitory pathways within the cortico-limbic-pontocerebellar network, whether from ALS, MS, or stroke, produces sudden, context-incongruent affective bursts [16,17]. Cerebellopontine strokes can produce comparable dysmetria of emotional expression, with preserved mood but loss of regulatory control, highlighting the shared circuitry underlying diverse etiologies [18]. Together, these findings demonstrate that PBA reflects a failure of coordinated network modulation - rather than isolated cortical injury - uniting classical clinico-anatomical descriptions with modern connectomic evidence.

Electrophysiologic studies reveal aberrant cortical activation preceding emotional outbursts, supporting the concept of impaired predictive gating and loss of top-down inhibitory control [17]. Complementing these findings, neurochemical studies demonstrate that PBA arises from disturbances across serotonergic and glutamatergic pathways. Serotonin provides tonic inhibition along fronto-cerebellar projections, and reduced serotonergic tone after injury correlates with symptom improvement following selective serotonin reuptake inhibitor (SSRI) therapy [19]. Conversely, excessive glutamatergic excitation via N-methyl-D-aspartate (NMDA) receptors contributes to emotional hyperreactivity, while sigma-1 receptor activation appears to restore excitatory-inhibitory balance [20]. Dextromethorphan (DM), a sigma-1 agonist and NMDA antagonist, combined with quinidine (Q) to boost its bioavailability, has demonstrated robust efficacy across multiple neurologic etiologies [21].

Lesion-mapping in ALS and MS demonstrates that PBA severity correlates with degeneration of corticobulbar tracts and the anterior cingulate [22-24]. Loss of synchrony across this distributed network decouples emotional expression from subjective experience, a phenomenon termed emotional dysmetria, analogous to cerebellar motor ataxia [25]. Across multiple imaging and neuropathologic studies, disruption of fronto-pontine-cerebellar pathways and resulting excitatory-inhibitory imbalance consistently emerge as key mechanisms [26]. This convergence supports the unifying “network-disconnection” model originally proposed by Rabins and Arciniegas, wherein lesions at any level of the cortico-limbic-subcortico-thalamic-pontocerebellar axis produce the same expressive disinhibition [27]. By integrating classical release theories with modern connectomic data, this model positions PBA as a paradigmatic example of emotion as an emergent property of distributed neural control.

Clinical recognition and differentiation

Clinically, PBA presents as brief, stereotyped episodes of involuntary laughter or crying that are disproportionate to external stimuli and incongruent with mood. Episodes last seconds to minutes, terminate abruptly, and leave insight intact [28]. These paroxysms, first described by classical neurologists, reflect a transient loss of higher cortical control over the brainstem networks mediating affective expression. They are often accompanied by vegetative signs such as tearing, facial flushing, or salivation, indicating autonomic-somatic co-activation within the same expressive circuitry [12]. Together, these physiological observations underscore that PBA represents a motor-expressive rather than affective phenomenon, aligning with the concept of emotional incontinence rather than mood disturbance.

Distinguishing PBA from mood disorders remains the primary diagnostic challenge. Unlike major depression, PBA does not involve sustained dysphoria, anhedonia, or vegetative symptoms between episodes [28]. Crying spells occur abruptly and without the subjective sadness or cognitive features typical of depressive states. Patients frequently describe embarrassment and frustration at their inability to regulate expression, despite preserved emotional awareness and contextual responsiveness. This incongruence between outward display and inner feeling, recognized by early authors as the hallmark of expressive disinhibition [7-9], remains the key clinical clue differentiating PBA from affective illness.

Misdiagnosis of PBA remains common, particularly in post-stroke, dementia, and ALS populations, where episodes are often mistaken for depression or anxiety. This diagnostic confusion delays treatment and compounds social withdrawal and caregiver distress. Epidemiologic surveys estimate prevalence between 10% and 49% across neurological conditions [29]. The large multicenter PRISM study of over 2,500 patients found that approximately one-third exhibited PBA-like symptoms, yet fewer than 10% received a formal diagnosis or therapy [30]. This persistent under-recognition reflects both the syndrome’s paroxysmal nature and its historical conflation with mood disorders.

Standardized tools, such as the Center for Neurologic Study-Lability Scale (CNS-LS) and the Pathological Laughing and Crying Scale, enable objective quantification and should be incorporated into routine neurologic assessment [31]. Still, these scales primarily measure emotional lability rather than true incontinence of affect, underscoring the ongoing need for refined diagnostic criteria that capture the expressive disinhibition at PBA’s core [5,6].

Neuroimaging findings often complement, but do not define, the diagnosis of PBA. Lesions involving the corticobulbar tracts, anterior cingulate, orbitofrontal cortex, or cerebellar vermis are frequently observed but remain supportive rather than diagnostic [32]. Recent imaging and connectivity studies reinforce that these structural changes reflect dysfunction within a broader cortico-limbic-pontocerebellar network, rather than isolated focal damage [15]. Integrating this data with clinical observation enhances diagnostic precision and underscores that PBA is best conceptualized as a distributed expressive network disorder, where structural disconnection and impaired inhibitory gating jointly drive the phenotype [7,8].

Therapeutic advances and future directions

Therapeutic approaches to PBA aim to restore inhibitory balance within the cortico-limbic-pontocerebellar network, targeting the neural circuits of emotional expression rather than mood regulation. Historically, treatment evolved from empirical antidepressant use to rational, mechanism-based pharmacotherapy, directed at the disorder’s underlying neurochemistry. As PBA reflects expressive disinhibition driven by serotonergic and glutamatergic imbalance, modern therapy focuses on re-establishing top-down cortical inhibition and modulating excitatory drive within cerebellar and brainstem pathways [33,34].

Early pharmacologic management relied on antidepressants, particularly SSRIs and tricyclic antidepressants (TCAs), which were shown in small, controlled trials to reduce the frequency and intensity of uncontrollable emotional episodes [33]. Their benefit is attributed to enhanced serotonergic tone along fronto-cerebellar pathways, thereby restoring inhibitory control over the faciovocal apparatus, rather than treating mood per se. Despite meaningful symptom improvement in many patients, responses were inconsistent, and onset was often delayed, prompting the development of mechanism-based agents designed specifically for PBA’s neurochemical signature.

DM/Q represents the first therapy developed specifically for PBA. Validated in randomized controlled trials and long-term open-label extensions [34-36], DM/Q exerts complementary actions that directly counter the excitatory-inhibitory imbalance underlying expressive disinhibition. DM functions as an NMDA receptor antagonist and sigma-1 receptor agonist, modulating both glutamatergic overactivity and neuronal signaling stability, while Q inhibits hepatic metabolism of DM, markedly enhancing its bioavailability. By restoring physiological inhibition within cortico-limbic-pontocerebellar circuits, DM/Q reduces the spontaneous discharge of the brainstem laughter- and crying-centers, thereby normalizing affective expression rather than mood.

Across multiple randomized and open-label studies, DM/Q has consistently demonstrated approximately a 50% reduction in PBA episode frequency and severity among patients with ALS and MS, accompanied by meaningful improvements in social functioning and quality of life [34-36]. Benefits are typically observed within the first two weeks of therapy and are maintained with long-term use, with dizziness and diarrhea being the most common, mild adverse effects. Comparable efficacy has been documented in TBI, cerebellar lesions, and progressive multifocal leukoencephalopathy, supporting its cross-etiologic utility and reinforcing that PBA reflects a shared network dysfunction rather than disease-specific pathology [34-36].

Comprehensive management of PBA extends beyond pharmacologic intervention. Behavioral and educational strategies remain crucial to mitigating PBA’s psychosocial impact. Educating patients and caregivers that these emotional outbursts stem from a neurologic disinhibition rather than a psychiatric instability reduces stigma, enhances adherence, and restores a sense of control. While formal behavioral therapies have not yet been validated in large trials, smaller studies and clinical reports support the use of trigger recognition, relaxation and breathing techniques, and cognitive reframing to diminish distress and improve self-efficacy [27,37]. Integrating neurologic, psychological, and rehabilitative perspectives allows treatment to address both the expressive and experiential dimensions of recovery, aligning with patient-centered models of neuropsychiatric care.

Recent systematic reviews confirm that SSRIs and DM/Q remain the most effective first-line options for PBA across neurologic conditions, with multimodal strategies recommended when depressive comorbidity is present [38]. Emerging data continues to highlight the profound social and quality-of-life consequences of untreated PBA. In a recent study, Finegan et al. (2025) demonstrated that emotional incontinence in primary lateral sclerosis correlates strongly with social withdrawal, caregiver burden, and diminished self-esteem, reinforcing that effective recognition and treatment carry psychosocial as well as neurologic significance [39].

These findings underscore the need for standardized screening, timely pharmacologic intervention, and the integration of patient-centered education into neurological care. As a paradigmatic disorder of network disinhibition, PBA illustrates how understanding the neural regulation of expression can guide both symptom relief and broader insights into brain-emotion connectivity.

Table 1 summarizes principal therapeutic approaches for PBA, linking mechanistic targets with clinical evidence.

**Table 1:** Pharmacologic and Behavioral Strategies for Pseudobulbar Affect The table summarizes principal therapeutic approaches for PBA, linking mechanistic targets with clinical evidence. Abbreviations: SSRI, selective serotonin reuptake inhibitor; TCA, tricyclic antidepressant; NMDA, N-methyl-D-aspartate; DM/Q, dextromethorphan/quinidine; GABA, gamma-aminobutyric acid

Therapeutic Class	Example Agents	Primary Mechanism	Representative Evidence	Clinical Notes
SSRIs	Sertraline, Citalopram	Enhance serotonergic inhibition of cortico-limbic circuits	[19,22,23,34]	Useful for mild PBA or comorbid depression; delayed onset
TCAs	Amitriptyline, Nortriptyline	Serotonin and norepinephrine reuptake inhibition	[33,34]	Efficacious but limited by anticholinergic effects
NMDA/sigma-1 modulators	DM/Q	Restores excitatory-inhibitory balance	[17,19,20,34-36]	Rapid and sustained benefit across neurologic etiologies
Anticonvulsants	Valproic acid	Stabilizes membrane excitability and GABA activity	[35]	Limited evidence; case-level support
Behavioral therapy and education	Trigger recognition, relaxation training	Reduces psychosocial impact	[27,37]	Adjunctive to pharmacotherapy

Discussion

The accumulated evidence positions PBA, historically termed emotional incontinence, as a model disorder for understanding the neural regulation of emotional expression, rather than the experience of emotion itself [5-8]. Across ALS, MS, stroke, TBI, and cerebellar disease, convergent findings reveal a shared breakdown of cortico-limbic-pontocerebellar connectivity that normally synchronizes expressive motor output with the internal emotional state [7,8,13-15]. The result is a disjunction between feeling and expression: laughter or crying released from voluntary control, but unaccompanied by congruent affect [6-8].

This distinction between emotional experience and expressive control, first articulated by Magnus, Bechterew, and Wilson [5-8], anticipates modern network neuroscience. Structural and functional imaging confirm that lesions or degenerative processes affecting corticobulbar tracts, cerebellar vermis, and anterior cingulate cortex yield the same phenotype of disinhibited affective display [7,8,13-15]. These data validate classical notions of “release phenomena” within a modern connectomic framework, uniting historical phenomenology with contemporary neurobiology.

Beyond elucidating pathophysiology, PBA provides a window into the interaction between neural circuitry, neurotransmitter dynamics, and social functioning [16-21]. Serotonergic and glutamatergic modulation restores inhibitory tone, demonstrating that targeted neurochemical intervention can re-establish emotional homeostasis [19-21]. At the same time, the persistence of stigma and misdiagnosis underscores the gap between scientific understanding and clinical practice. Integrating neurobiologic insight with patient education reframes PBA as a neurologic - not psychiatric - syndrome, promoting both empathy and adherence [27,31,33-36,38,39].

These classical observations of vegetative and facial co-activation, later confirmed electrophysiologically [12], further illustrate that PBA is the release of an evolutionarily conserved expressive network encompassing somatic and autonomic components of emotion [5-8,12-14]. In this sense, PBA is to affect what ataxia is to movement - an error of coordination rather than intent [7,8,13,14].

Persistent underdiagnosis remains the foremost challenge. Large-scale studies such as PRISM demonstrate that most individuals exhibiting symptoms of PBA remain untreated [4]. Routine screening with validated tools like the CNS-LS [31], paired with early initiation of pharmacologic and behavioral interventions [27,33-36], can meaningfully improve function and reduce caregiver burden [39].

By situating PBA within the broader continuum of network disorders, the syndrome serves as a paradigmatic example of how brain disconnection manifests as emotional dysregulation and how restoring network integrity can restore dignity and social connection [5-8,13,14,39].

Limitations

This review integrates data from heterogeneous sources with variable methodology and diagnostic rigor. Lack of uniform outcome metrics precluded meta-analytic synthesis. Nevertheless, consistent convergence across imaging, pharmacologic, and clinical evidence lends confidence to the unified network model. Future large-scale systematic reviews may better quantify prevalence, treatment response, and long-term outcomes.

## Conclusions

PBA represents a distinct, biologically grounded disorder of emotional expression, arising from disrupted inhibitory-excitatory balance within cortico-limbic-pontocerebellar circuits. Once dismissed as a minor behavioral complication, it now stands as a window into the neural orchestration of emotion and the therapeutic potential of network restoration.

Bridging the insights of early neurologists with modern imaging and neuropharmacology, this review reframes PBA as a form of “emotional ataxia” - a failure of expressive precision rather than of feeling itself. Effective, mechanism-based treatments demonstrate that modulation of these pathways can normalize expression and improve quality of life. Embedding PBA recognition into routine neurologic care will ensure that this once-misunderstood condition is met not with confusion or stigma, but with compassion and evidence-based treatment.
